# Supplementary material for: Human Semaphorin-4A drives Th2 responses by binding to receptor ILT-4
Source: Nat Commun. 2018 Feb 21;9:742. doi: 10.1038/s41467-018-03128-9 (PMC5821868; doi:10.1038/s41467-018-03128-9)
Supplement: Supplementary file 1 — Supplementary Information [file 41467_2018_3128_MOESM1_ESM.pdf]

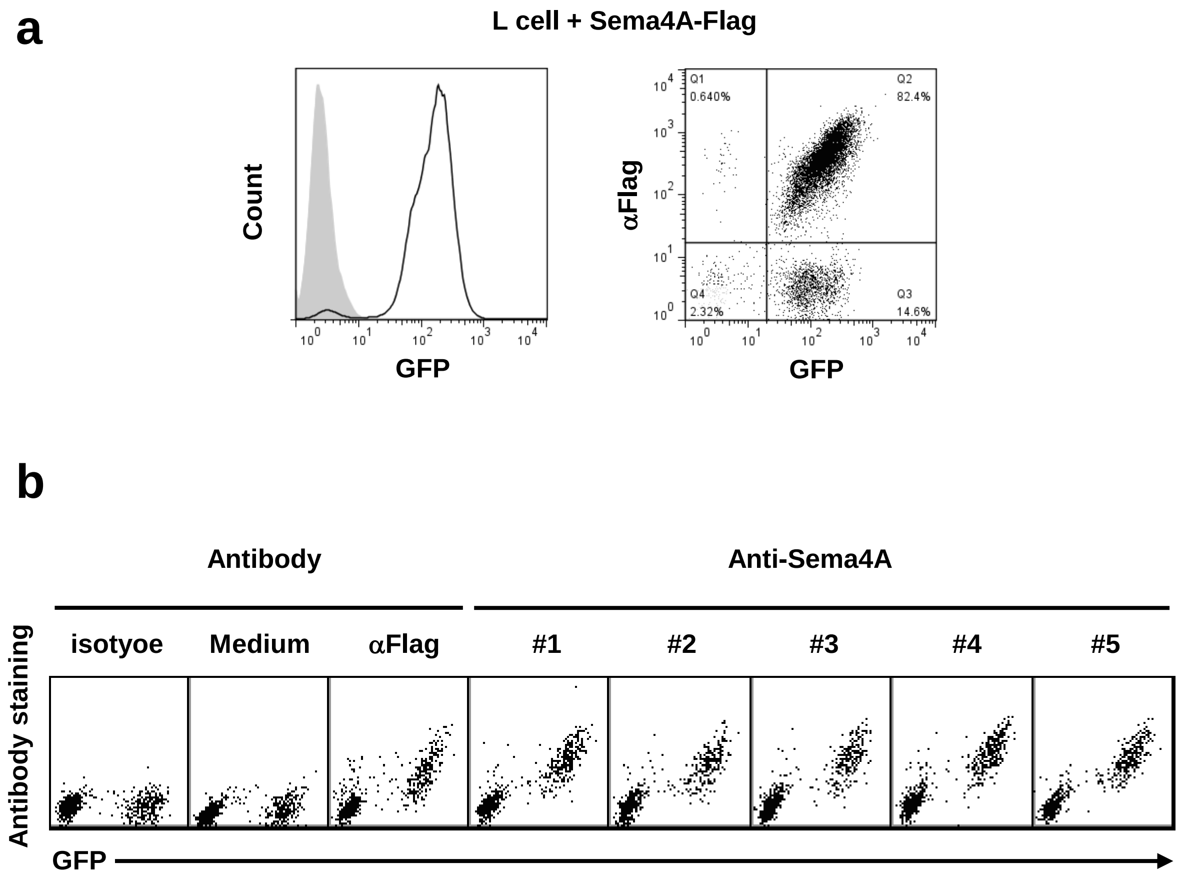

Supplemental figure 1. Generation and screening of the monoclonal antibodies against human Sema4A (a) cDNA of human Sema4A extracellular domain was cloned and over expressed in a mouse L cell line using a pMXS-GFP-Flag vector. A human Sema4A-transfected L cell line was shown to be GFP^+ and $Flag^+$ by FACS. (b) $Sema4A^+GFP^+Flag^+$L cells mixed with parental L cells at a ratio of 1:1 were stained with different clones of monoclonal antibodies (mAbs) against human Sema4A generated by our lab followed by staining with fluorescent-labeled anti-mouse Immunoglobulin G (mIgG) antibody. The isotypes served as the controls. Parental L cells were negative controls for the human Sema4A-transfected L cell line. Anti-Flag antibody was used as positive controls for anti- Sema4A antibodies.

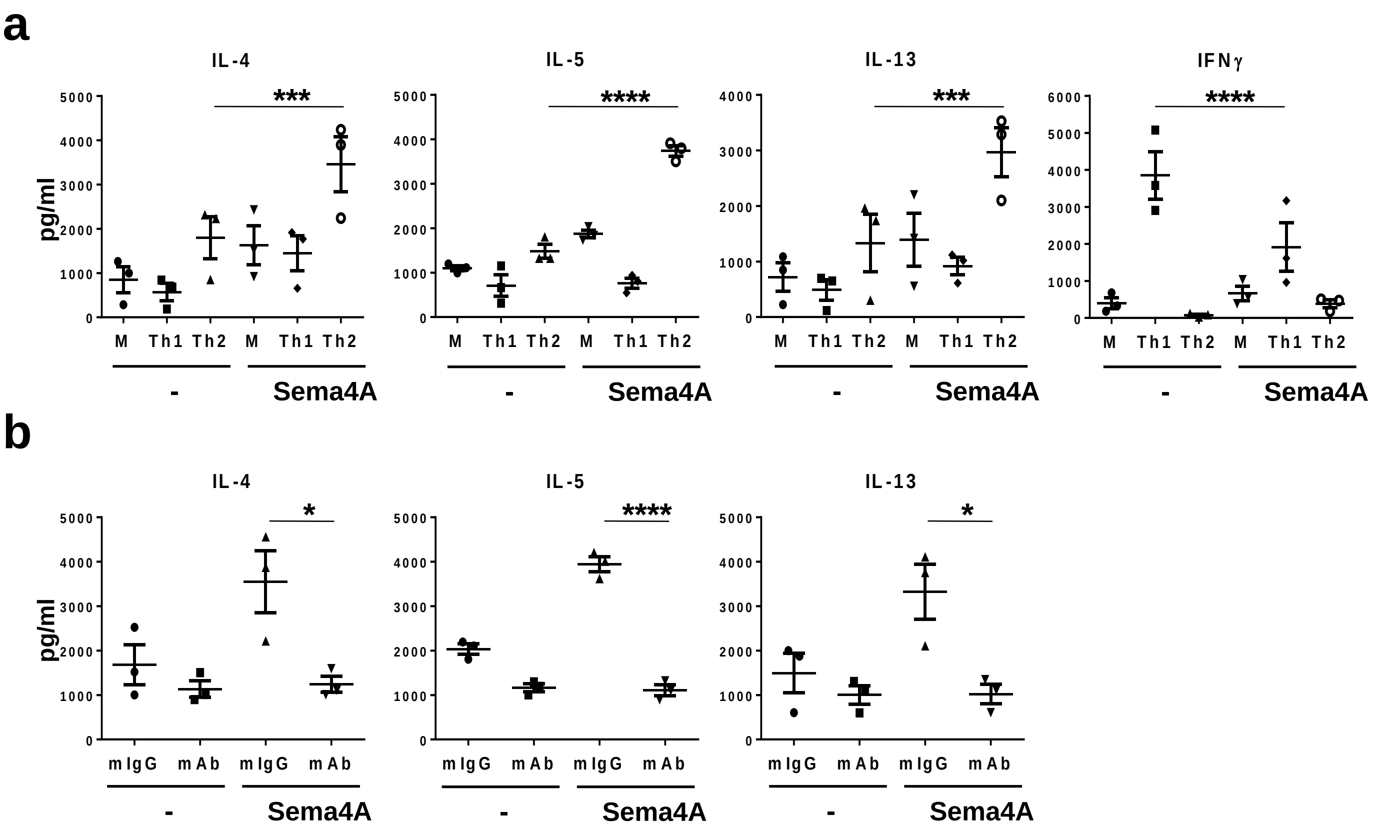

Supplemental figure 2. Sema4A enhances Th2 cytokine production of CRTH2^{+} Th2 memory cells. (a-b). The purified CRTH2^{+}Th2 memory cells were cultured on parental L cells or Sema4A-expressing L cells pre-coated with a sub-optimal dose of anti-CD3 mAb plus anti-CD28 mAbs or in Th1- or Th2- skewing conditions (a); or in Th2-skewing condition with anti-Sema4A mAb or mouse Immunoglobulin G (mIgG) (b) for 7 days. Cultured T cells were harvested and re-stimulated with immobilized anti-CD3 plus anti-CD28 for 24 hours before the measurements of cytokines in the supernatants by ELISA. Each dot represents one donor. Data represents one of five independent experiments. T student tests were used for statistical analysis, P value was as shown, *$p < 0.05$, **$p < 0.01$, ***$p < 0.001$, ****$p < 0.0001$.

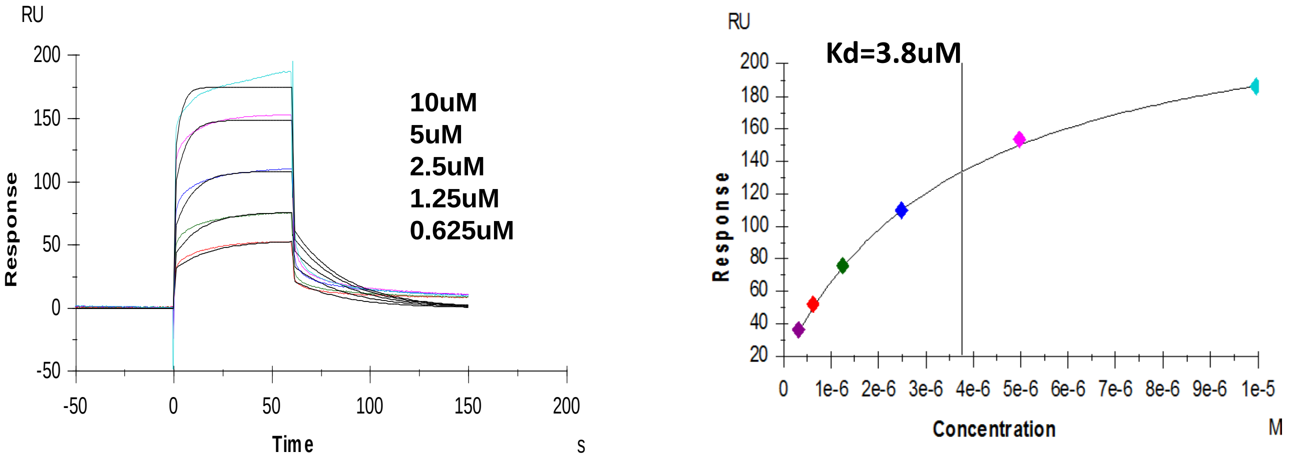

Supplemental figure 3. Surface Plasmon Resonance of Sema4A-Fc binding to ILT-4-Fc. ILT-4-Fc diluted from 10M to 0.625 M at 2X fold ratio was injected through the flow cell with Sema4A-Fc immobilized on the sensor chip. As control, ILT-4-Fc samples were injected through a flow cell without Sema4A-Fc. Binding curve represents the increase in flow cells containing Sema4A-Fc as compared with control flow cells.

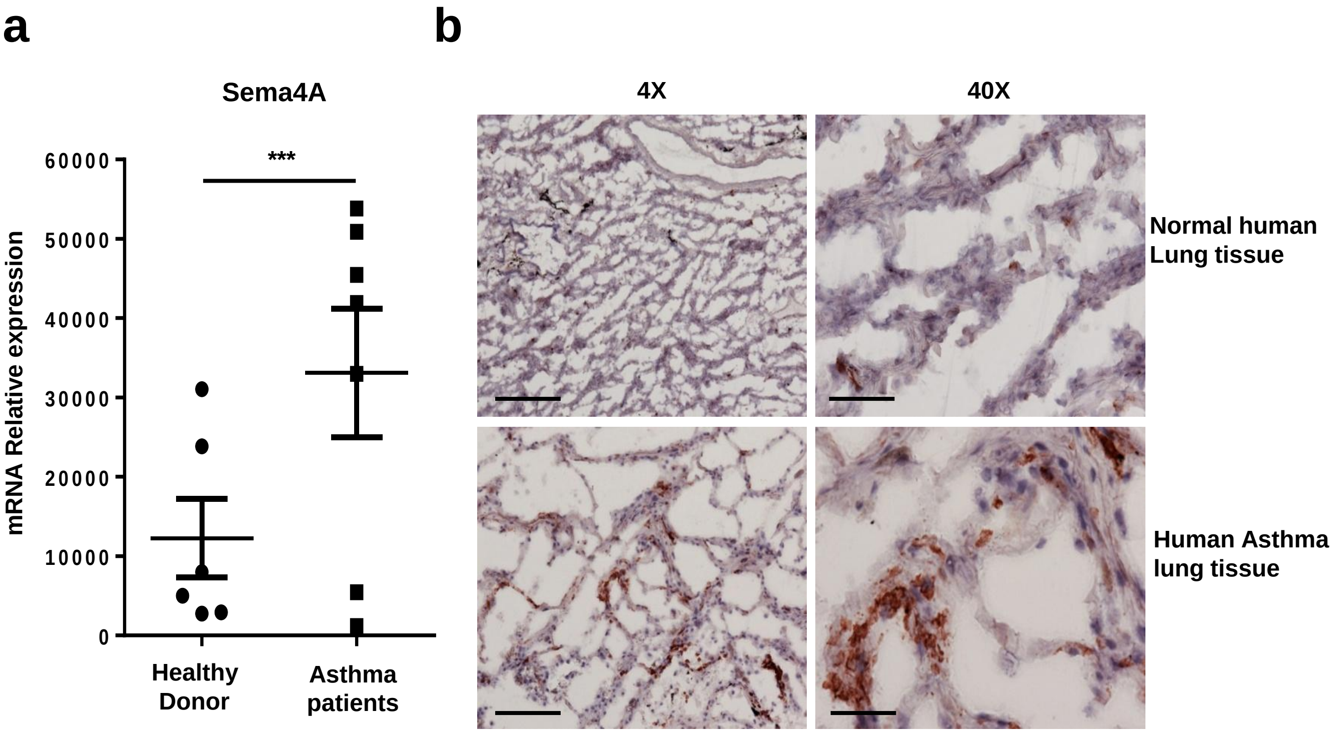

Supplemental figure 4. Sema4A is specifically over expressed in human asthma lung tissue. (a) Total RNA from lung tissue of healthy donor or asthma patient was isolated and reverse transcribed into cDNAs for use as templates. With a specific primer for hSema4A (ABI), Q-PCR analysis was conducted. The relative expression of hSema4A is shown (P<0.001)(T student tests). Each dot represents one donor. The short line in each group represents the average expression. More than six individual samples were analyzed. (b).Human normal and asthma lung tissue sections were stained with anti-Sema4A mAb followed by HRP conjugated goat anti-mouse antibody. Magnifications used for pictures are ×4 (left) with scale bar representing 500μm and ×40 (right) with scale bar representing 50μm. More than two individual samples were analyzed.

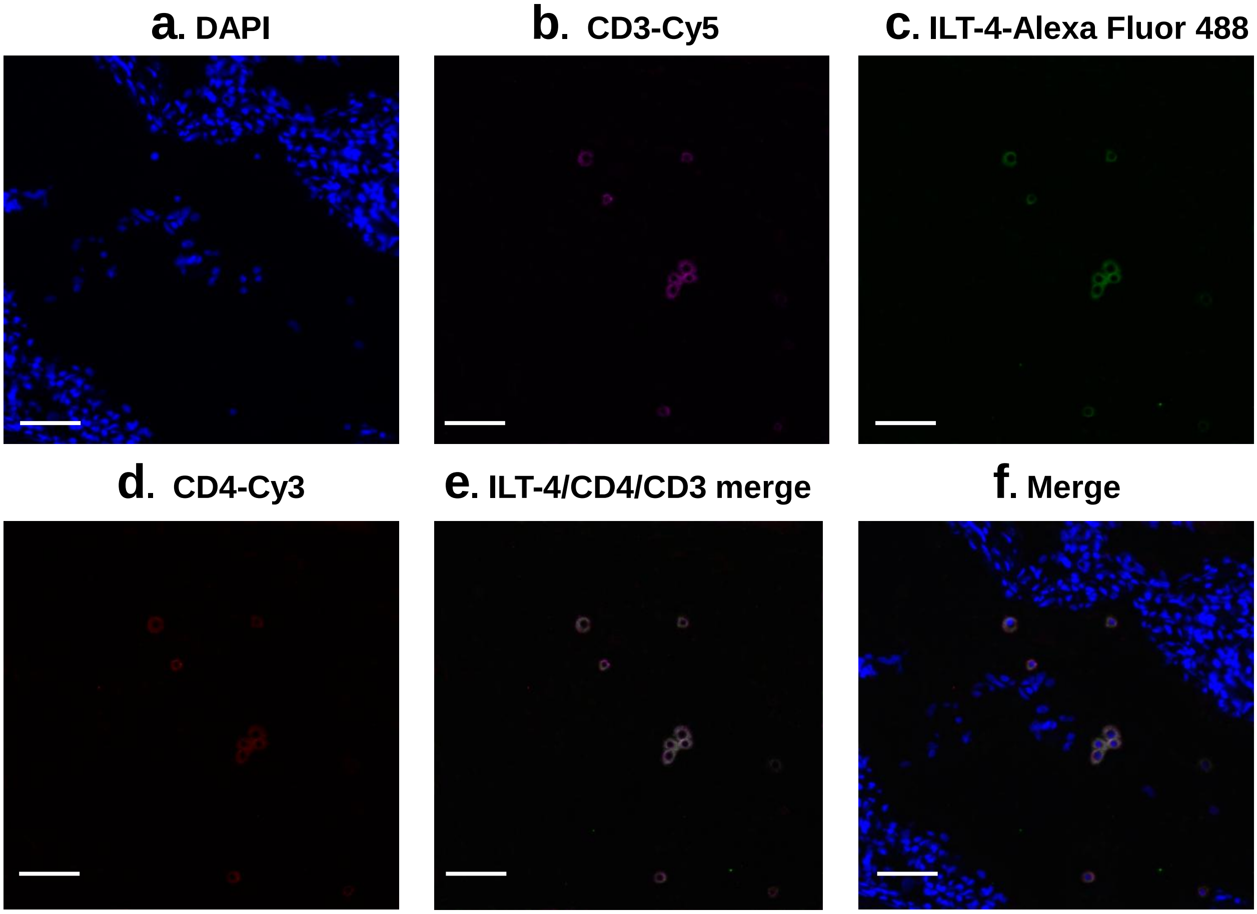

Supplementary figure 5. Colocalization of endogenous CD3, CD4 and ILT4 in infiltrated T cells from human asthma lung tissue. Confocal microscopy of infiltrated T cells from human asthma lung tissue. DAPI served as the nuclei marker (blue, a). CD3 was stained with Cy5 conjugated mouse anti-CD3 monoclonal antibody (1:100) (LS-C351533, LifeSpan BioSciences Inc.) (Cyan, b), while ILT4 was stained with Rabbit anti-LILRB2 (ILT4) polyclonal antibody (1:200) (PAB153Hu01, Cloud-Clone Corp.), followed by Alexa Fluor 488 goat anti-rabbit secondary antibody (1:1000, Cat. ab150077, abcam) (green, c). Furthermore CD4 was stained with Rat anti-CD4 monoclonal antibody (1:200) (ab34276, abcam), followed by Cy3 conjugated donkey anti-Rat secondary antibody (1:1000, Cat. AP189C, EMD Millipore Corp.) (red, d). (e) showed the merged image of CD3, CD4 and ILT4, while (f) showed the merged image of CD3, CD4 and ILT4 with DAPI. The Scale bars represent 50 µm.

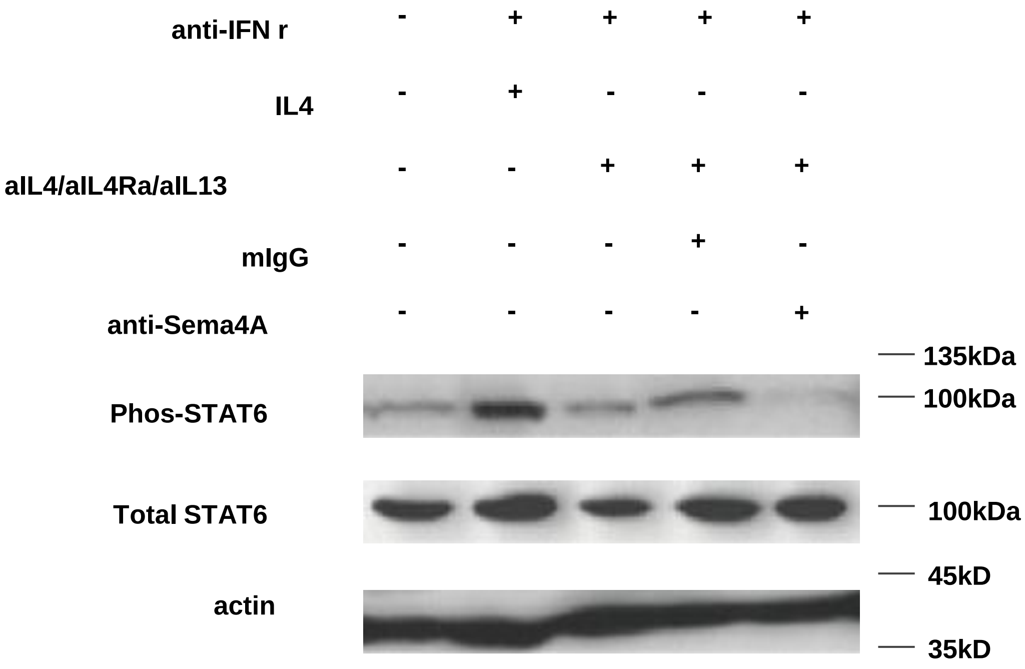

Supplementary figure 6. Sema4A enhances activation of SATA6 on pre-stimulated CD4+T cells. Purified naïve CD4+ T cells were cultured with CD32+Sema4A+L cells in conditions of Medium, Th2 (anti-IFNγ, IL-4), Th0(anti-IFN γ, anti-IL4, anti-IL4Rα, anti-IL13), Th0 plus mouse Immunoglobulin G (mIgG) and Th0 plus anti-Sema4A as indicated accordingly. After 9 days culture, activated T cells were harvested, washed with PBS twice, and re-stimulated with immobilized OKT3 plus anti-CD28 for 19 hours. Activated STAT6 were detected by Western blot. Actin serves as an internal control. Data represents one of two independent experiments.

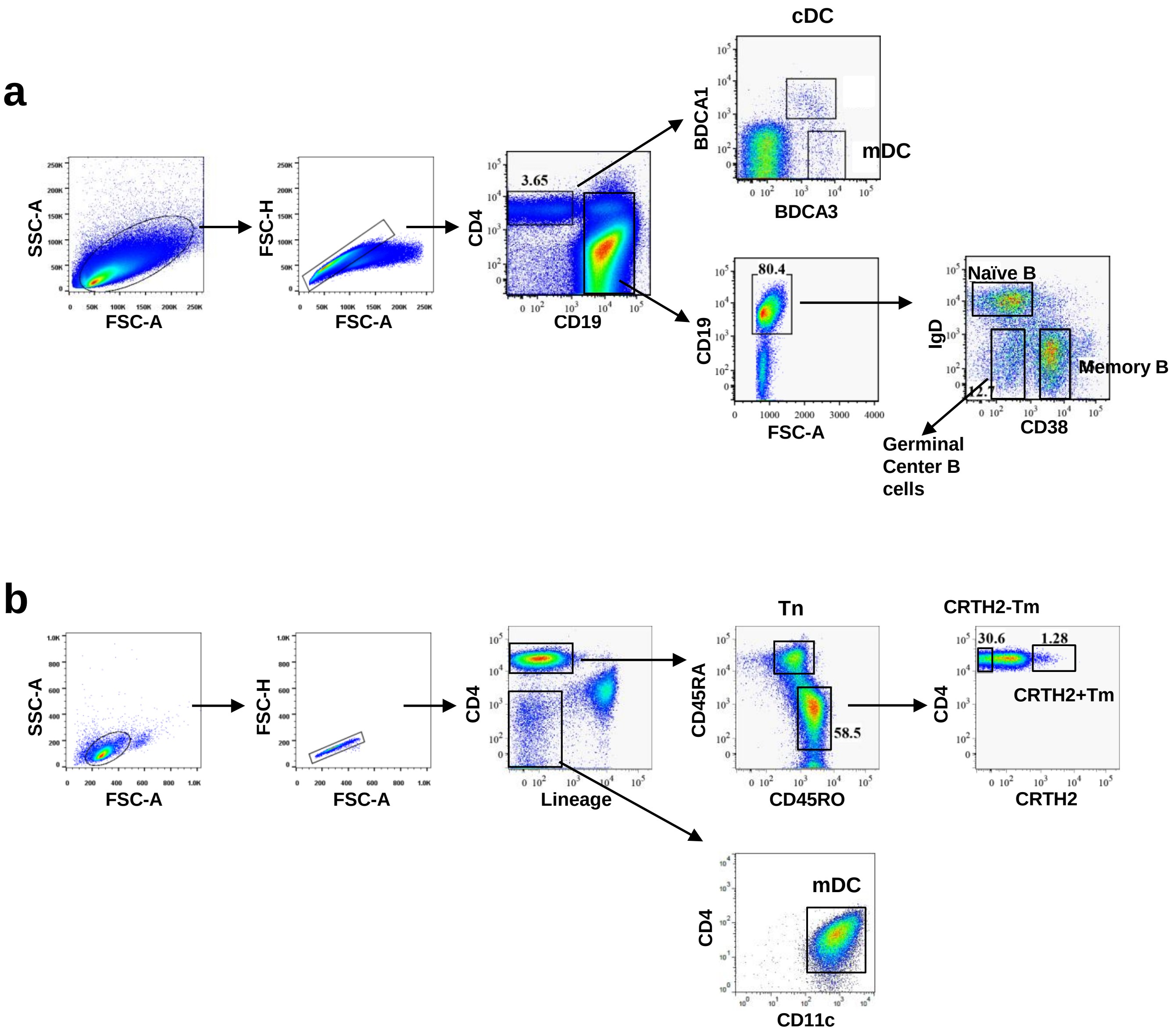

Supplementary Figure 7. Gating strategies used for cell sorting. (a) Gating strategy to sort tonsil B cells and Dcs for FACS staining on Fig 1 C left 2 panels; (b) Gating strategy to sort CD4^{+} native T, CD4^{+}CD45RO^{+}CRTH2^{+} Th2 memory T, CD4^{+}CD11c^{+} mDC cells from human PBMC for FACS staining on Fig 1c right 2 panels, Fig 4a, Fig 5b, c, d; and for in vitro cultures presented on Fig 2, 3, 6, Fig S2 and Fig S6.

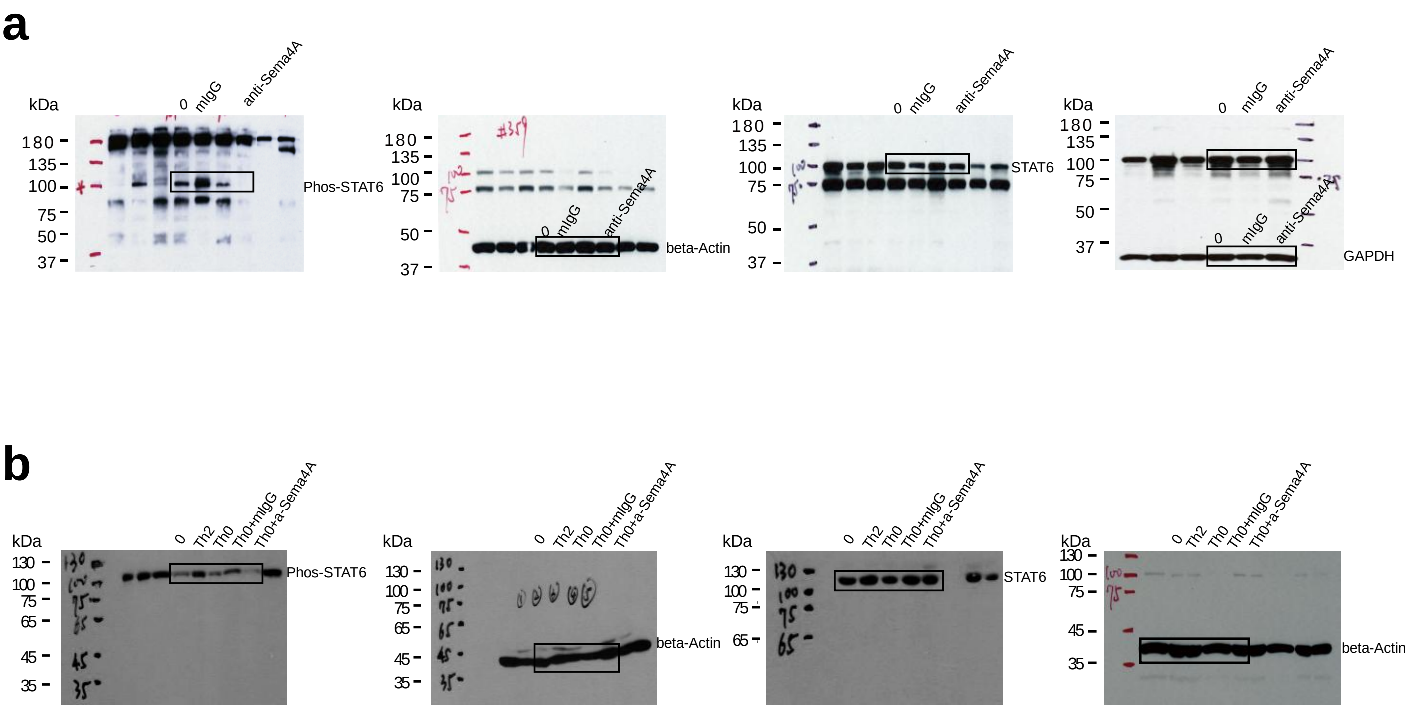

Supplementary Figure 8. Original data of immunoblots. (a) Immunoblot data corresponding to Fig. 3e. (b) Immunoblot data corresponding to Fig. S6.
